# Characterization of four cluster A1 *Mycobacterium* phage genomes, Applejack, Hermia, LilBib, and QTRlifeCrisis

**DOI:** 10.1128/mra.00244-25

**Published:** 2025-09-26

**Authors:** Noah E. Adelman, Leah Cromarty, Sonja M. Francis, Vejune E. Griciute, Jason N. Hart, Abigail Hoffpauir, Ryan Koss, Ronit Kumar, Melisa N. Matonsi, Emma Morrison, Grace Paiement, Autumn Perley, Adrian Plichta, Liliana Rodrigues, Melody N. Neely, Caitlin S. Wiafe-Kwakye, Sally D. Molloy

**Affiliations:** 1 The Honors College, University of Maine 6250, Orono, Maine, USA; 2 Molecular and Biomedical Sciences, University of Maine 6250, Orono, Maine, USA; University of Pittsburgh School of Medicine, Pittsburgh, Pennsylvania, USA

**Keywords:** bacteriophage, actinobacteria, genome

## Abstract

Four novel cluster A1 siphoviruses, Applejack, Hermia, LilBib, and QTRlifeCrisis, were isolated on *Mycobacterium smegmatis*. Their complete genome sequences are 49,633–50,325 bp in length and encode 80–91 protein-coding genes. Applejack and LilBib encode metallophosphatases containing inteins, and Applejack encodes a DNA methylase that contains an out-of-frame HNH endonuclease.

## ANNOUNCEMENT

Actinobacteriophage are diverse viruses that infect Actinobacteria, including non-pathogenic and pathogenic *Mycobacterium* species (1–6). Isolating and characterizing novel phages provides insight to phage evolution and opportunities to improve treatments for antibiotic-resistant mycobacterial infections (5, 7). Phages Applejack, Hermia, LilBib, and QTRlifeCrisis were isolated on the host *Mycobacterium smegmatis* MC^2^155 using direct and enrichment isolation procedures (Table 1). Soil extracts were prepared in 7H9 top agar, incubated for 1–2 h at 30°C and filtered on a 0.22-µM filter. For direct isolations, soil extracts were inoculated into *M. smegmatis*, incubated for 15 min and plated in 7H9 top agar onto L-agar plates. For enrichment isolations, soil extracts were inoculated with *M. smegmatis*, incubated for 2 d at 30°C with shaking before filtering onan 0.22-µM filter. Serially diluted extracts were plated with *M. smegmatis* and 7H9 top agar onto L-agar plates. Plates were incubated for 2daysd at 30°C and screened for plaques. Multiple rounds of standard plaque assays were performed to purify plaques (Table 1) (8). Hermia and LilBib consistently formed 4-mm turbid plaques. QTRlifeCrisis and Applejack plaques vary in size and have clear centers with turbid rings (Table 1). Transmission electron microscopy revealed particle morphologies consistent with *Siphoviridae* (Table 1).

**TABLE 1:** Phage and genome characteristics

Isolation data	Applejack	Hermia	LilBib	QTRlifeCrisis
Soil GPS coordinates	44.898789 N, 68.666355 W	44.896201 N, 68.672347 W	44.5549 N, 68.4206 W	44.896201 N, 68.672347 W
Soil isolation location	Orono, ME	Orono, ME	Orono, ME	Old Town, ME
Isolation procedure	Enrichment	Enrichment	Direct	Direct
Rounds of plaque assays to purify plaques	3	4	6	5
Plaque morphology	2–7 mm diameter, clear center turbid ring	4 mm diameter, turbid	4 mm diameter, turbid	2–4 mm, clear center turbid ring
Particle morphology and average particle dimensions (± the standard error)				
Morphology	Siphoviridae	Siphoviridae	Siphoviridae	Siphoviridae
Tail length (nm)	167 ± 2.03	171 ± 3.11	201 ± 2.26	199 ± 2.12
Capsid diameter (nm)	69.7 ± 0.724	72.3 ± 0.753	69.6 ± 1.01	72.4 ± 0.733
Number of particles measured	5	5	5	5
Sequencing and genome characteristics				
Genome length	49,791	49,670	50,325	49,633
GC content	63.5%	63.9%	63.8%	63.7%
Number 250 bp single-end reads	191,866	192,615	191,557	193,004
Fold coverage	708	836	625	928
Number of protein-coding genes	91	84	80	86
Number of tRNAs	0	0	0	0

Phage genomic DNA was isolated from high-titer lysates by phenol chloroform extraction (9) and prepared for sequencing using the Kapa Hyper Plus DNA library kit (Roche, South San Francisco, CA). Genomes were sequenced on an Illumina NovaSeq 6000 platform, and fold coverage is reported in Table 1. Newbler v.2.9 and Consed v29 were used to assemble genomes and check for completeness (10–12). The four genomes were 49,633–50,325 bp in length, have 63% GC content, and genome ends defined by 10 bp (CGGATGGTAA) 3′ overhangs (Table 1). They share >35% gene content with other cluster A mycobacteriophages in the Phamerator database (Actino_Draft v.589), and were assigned to subcluster A1 (2, 13).

**Fig 1:**
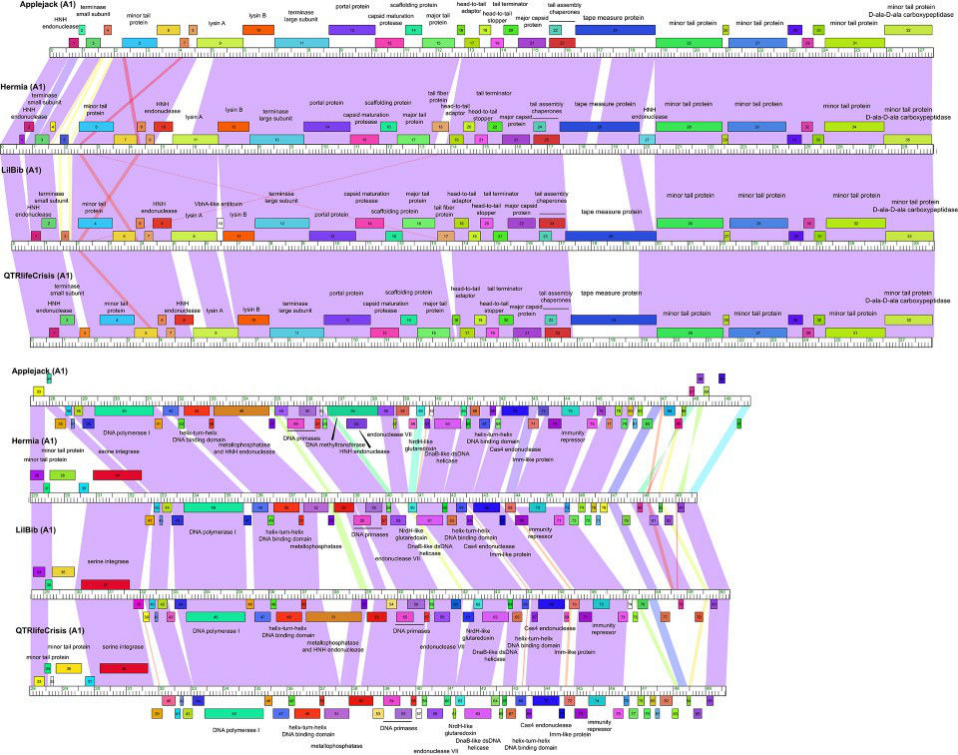
Genome map of cluster A1 *Mycobacterium* phages Applejack, Hermia, LilBib, and QTRlifeCrisis. The genome coordinates are represented by the ruler in units of kilobase pairs. The colored boxes above and below the ruler represent genes transcribed in the forward and reverse directions, respectively, and gene or gene product (gp) number is indicated within the box. Genes were assigned to a family using Phamerator in the Actino_draft database, and different families are indicated by colors (13). Colored shading between the genomes indicates nucleotide identity with violet of the color spectrum indicating the highest nucleotide identity. Predicted functions are centered above or below forward and reverse transcribed genes, respectively.

Genomes were auto-annotated using Glimmer v.3.02 and GeneMark v.2.5 embedded in DNAMaster v.5.23.6 and PECAAN (https://blog.kbrinsgd.org/) (14, 15). Translational starts were manually determined based on inclusion of coding potential and conservation across homologs using Starterator (http://phages.wustl.edu/starterator/), BLASTP, and GeneMark.hmm output (14, 16). Putative gene functions were predicted using BLASTP and HHpred (16, 17). Transmembrane domains were predicted using Deep TMHMM (18). No tRNAs were detected using Aragorn v.1.2.38 and tRNAScan-SE2.0 (19, 20). The genomes encode 80–91 protein-coding genes (Table 1). Left arms encode forward-transcribed structural and assembly genes (Fig. 1). Right arms encode reverse-transcribed genes including DNA polymerase I, an immunity repressor, and overlapping DNA primases (Fig. 1). Hermia, LilBib, and QTRlifeCrisis encode a serine integrase that is absent in Applejack.

All the genomes encode multiple homing endonucleases and metallophosphatases. However, Applejack and LilBib metallophosphatases (gp48 and 51) contain an intein with high probability HHpred alignment to the *Pyrococcus furiosis* intein PI-Pfu (Fig. 1) (21). This intein includes a HINT domain for protein splicing and a homing endonuclease domain with two LAGLIDADG motifs, components of the active site (21, 22). The function of the intein is unknown but potentially provides a mechanism of post-translational regulation (23). Applejack also has a DNA methylase (gp54) with an out-of-frame HNH endonuclease (gp55). An HNH endonuclease is found upstream of the small subunit terminase in all genomes and likely plays a role in genome packaging (24).

## Data Availability

Applejack, Hermia, LilBib, and QTRlifeCrisis genomes are available at GenBank with the Accession numbers PP978873.1, PP750959.1, PP750957.1, and PP758914.1 and the Sequence Read Archive (SRA) numbers SRX24123897, SRX24123887, SRX24123889, and SRX24123894, respectively.

## References

[1] Pope WH, Mavrich TN, Garlena RA, Guerrero-Bustamante CA, Jacobs-Sera D, Montgomery MT, Russell DA, Warner MH, Hatfull GF. 2017. Bacteriophages of Gordonia spp. Display a spectrum of diversity and genetic relationships. mBio 8:4. doi:10.1128/mBio.01069-17 PMC5559632 28811342

[2] Pope WH, Jacobs-Sera D, Russell DA, Peebles CL, Al-Atrache Z, Alcoser TA, Alexander LM, Alfano MB, Alford ST, Amy NE, et al.. 2011. Expanding the diversity of mycobacteriophages: insights into genome architecture and evolution. PLoS One 6:e16329. doi:10.1371/journal.pone.0016329 21298013 PMC3029335

[3] Pope Welkin H, Anders KR, Baird M, Bowman CA, Boyle MM, Broussard GW, Chow T, Clase KL, Cooper S, Cornely KA, et al.. 2014. Cluster M mycobacteriophages Bongo, PegLeg, and Rey with unusually large repertoires of tRNA isotypes. J Virol 88:2461–2480. doi:10.1128/JVI.03363-13 24335314 PMC3958112

[4] Jacobs-Sera D, Abad LA, Alvey RM, Anders KR, Aull HG, Bhalla SS, Blumer LS, Bollivar DW, Bonilla JA, Butela KA, et al.. 2020. Genomic diversity of bacteriophages infecting Microbacterium spp. PLoS One 15:e0234636. doi:10.1371/journal.pone.0234636 32555720 PMC7302621

[5] Hatfull GF. 2022. Mycobacteriophages: from petri dish to patient. PLoS Pathog 18:e1010602. doi:10.1371/journal.ppat.1010602 35797343 PMC9262239

[6] Cresawn SG, Pope WH, Jacobs-Sera D, Bowman CA, Russell DA, Dedrick RM, Adair T, Anders KR, Ball S, Bollivar D, et al.. 2015. Comparative genomics of cluster O mycobacteriophages. PLoS One 10:e0118725. doi:10.1371/journal.pone.0118725 25742016 PMC4351075

[7] Dedrick RM, Guerrero-Bustamante CA, Garlena RA, Russell DA, Ford K, Harris K, Gilmour KC, Soothill J, Jacobs-Sera D, Schooley RT, Hatfull GF, Spencer H. 2019. Engineered bacteriophages for treatment of a patient with a disseminated drug-resistant Mycobacterium abscessus. Nat Med 25:730–733. doi:10.1038/s41591-019-0437-z 31068712 PMC6557439

[8] Poxleitner M, Pope W, Jacobs-Sera D, Sivanathan V, Hatfull G. 2018. Phage discovery guide. Howard Hughes Medical Institute, Chevy Chase, MD.

[9] Green MR, Sambrook J. 2017. Isolation of high-molecular-weight DNA using organic solvents. Cold Spring Harb Protoc 2017:pdb.prot093450. doi:10.1101/pdb.prot093450 28373491

[10] Russell DA. 2017. Sequencing, assembling, and finishing complete bacteriophage genomes, p 109–125. In Bacteriophages: methods and protocols. Vol. 3. Springer. 10.1007/978-1-4939-7343-9_9 29134591

[11] Hatfull GF, Jacobs-Sera D, Lawrence JG, Pope WH, Russell DA, Ko C-C, Weber RJ, Patel MC, Germane KL, Edgar RH, et al.. 2010. Comparative genomic analysis of 60 mycobacteriophage genomes: genome clustering, gene acquisition, and gene size. J Mol Biol 397:119–143. doi:10.1016/j.jmb.2010.01.011 20064525 PMC2830324

[12] Gordon D, Green P. 2013. Consed: a graphical editor for next-generation sequencing. Bioinformatics 29:2936–2937. doi:10.1093/bioinformatics/btt515 23995391 PMC3810858

[13] Cresawn SG, Bogel M, Day N, Jacobs-Sera D, Hendrix RW, Hatfull GF. 2011. Phamerator: a bioinformatic tool for comparative bacteriophage genomics. BMC Bioinformatics 12:395. doi:10.1186/1471-2105-12-395 21991981 PMC3233612

[14] Besemer J, Borodovsky M. 2005. GeneMark: web software for gene finding in prokaryotes, eukaryotes and viruses. Nucleic Acids Res 33:W451–W454. doi:10.1093/nar/gki487 15980510 PMC1160247

[15] Delcher AL, Harmon D, Kasif S, White O, Salzberg SL. 1999. Improved microbial gene identification with GLIMMER. Nucleic Acids Res 27:4636–4641. doi:10.1093/nar/27.23.4636 10556321 PMC148753

[16] Altschul SF, Madden TL, Schäffer AA, Zhang J, Zhang Z, Miller W, Lipman DJ. 1997. Gapped BLAST and PSI-BLAST: a new generation of protein database search programs. Nucleic Acids Res 25:3389–3402. doi:10.1093/nar/25.17.3389 9254694 PMC146917

[17] Söding J, Biegert A, Lupas AN. 2005. The HHpred interactive server for protein homology detection and structure prediction. Nucleic Acids Res 33:W244–W228. doi:10.1093/nar/gki408 15980461 PMC1160169

[18] Hallgren J, Tsirigos KD, Pedersen MD, Almagro Armenteros JJ, Marcatili P, Nielsen H, Krogh A, Winther O. 2022. DeepTMHMM predicts alpha and beta transmembrane proteins using deep neural networks. Bioinformatics. doi:10.1101/2022.04.08.487609

[19] Lowe TM, Eddy SR. 1997. tRNAscan-SE: a program for improved detection of transfer RNA genes in genomic sequence. Nucleic Acids Res 25:955–964. doi:10.1093/nar/25.5.955 9023104 PMC146525

[20] Laslett D, Canback B. 2004. ARAGORN, a program to detect tRNA genes and tmRNA genes in nucleotide sequences. Nucleic Acids Res 32:11–16. doi:10.1093/nar/gkh152 14704338 PMC373265

[21] Ichiyanagi K, Ishino Y, Ariyoshi M, Komori K, Morikawa K. 2000. Crystal structure of an archaeal intein-encoded homing endonuclease PI-PfuI. J Mol Biol 300:889–901. doi:10.1006/jmbi.2000.3873 10891276

[22] Chevalier BS, Stoddard BL. 2001. Homing endonucleases: structural and functional insight into the catalysts of intron/intein mobility. Nucleic Acids Res 29:3757–3774. doi:10.1093/nar/29.18.3757 11557808 PMC55915

[23] Kelley DS, Lennon CW, Belfort M, Novikova O, SEA-PHAGES. 2016. Mycobacteriophages as incubators for intein dissemination and evolution. mBio 7:01537–16. doi:10.1128/mBio.01537-16 PMC5050341 27703073

[24] Kala S, Cumby N, Sadowski PD, Hyder BZ, Kanelis V, Davidson AR, Maxwell KL. 2014. HNH proteins are a widespread component of phage DNA packaging machines. Proc Natl Acad Sci USA 111:6022–6027. doi:10.1073/pnas.1320952111 24711378 PMC4000778

